# Self-reported medication side effects in an older cohort living independently in the community - the Melbourne Longitudinal Study on Healthy Ageing (MELSHA): cross-sectional analysis of prevalence and risk factors

**DOI:** 10.1186/1471-2318-10-37

**Published:** 2010-06-10

**Authors:** Jennifer A Thomson, Wei C Wang, Colette Browning, Hal L Kendig

**Affiliations:** 1 School of Primary Health Care, Monash University, Melbourne, Australia; 2 Faculty of Health Sciences, University Sydney, Sydney, Australia

## Abstract

**Background:**

Medication side effects are an important cause of morbidity, mortality and costs in older people. The aim of our study was to examine prevalence and risk factors for self-reported medication side effects in an older cohort living independently in the community.

**Methods:**

The Melbourne Longitudinal Study on Healthy Ageing (MELSHA), collected information on those aged 65 years or older living independently in the community and commenced in 1994. Data on medication side effects was collected from the baseline cohort (n = 1000) in face-to-face baseline interviews in 1994 and analysed as cross-sectional data. Risk factors examined were: socio-demographics, health status and medical conditions; medication use and health service factors. Analysis included univariate logistic regression to estimate unadjusted risk and multivariate logistic regression analysis to assess confounding and estimate adjusted risk.

**Results:**

Self-reported medication side effects were reported by approximately 6.7% (67/1000) of the entire baseline MELSHA cohort, and by 8.5% (65/761) of those on medication. Identified risk factors were increased education level, co-morbidities and health service factors including recency of visiting the pharmacist, attending younger doctors, and their doctor's awareness of their medications. The greatest increase in risk for medication side effects was associated with liver problems and their doctor's awareness of their medications. Aging and gender were not risk factors.

**Conclusion:**

Prevalence of self-reported medication side effects was comparable with that reported in adults attending General Practices in a primary care setting in Australia. The prevalence and identified risk factors provide further insight and opportunity to develop strategies to address the problem of medication side effects in older people living independently in the community setting.

## Background

Medication side effects also referred to as adverse drug reactions (ADRs) and/or adverse drug events (ADEs) are potentially associated with all medications and include side effects listed in product information, as well as medication-related side effects due to interactions, non-compliance [1,2], prescribing errors [3] and allergic reactions [4]. Medication side effects are an important cause of morbidity, mortality, costs [5-16], and dissatisfaction with care [16].

Prevalence of medication side effects depends on the surveillance system and setting [17]. Surveillance systems vary in case definitions, case finding, and the duration of monitoring [14]. Mostly, medication side effects have been examined in inpatient hospital settings and to a lesser extent in hospital outpatient or ambulatory clinic settings by chart review and self report. Information on medication side effects in the community is more limited and is mostly provided by national surveillance systems that rely on voluntary reporting and lack denominator data.

Similarly, potential risk factors for medication side effects have been examined mostly in hospital inpatient or outpatient or ambulatory clinic settings. The most consistently identified risk factors include older age, female gender, increasing number of medications, and increasing number of medical conditions/co-morbidities as well as specific co-morbidities [13,14,16,18]. Other risk factors include those related to compliance, comprehension and general patient knowledge, types of medications (in hospital settings such medications include antibiotics, anti-coagulants, digoxin, diuretics, hypoglycaemic, NSAIDs, chemotherapeutics, other cardiovascular medications and analgesics), hospitalisations, medical knowledge, multiple treating doctors, and multiple filling pharmacies [18-20].

Data from the 1994 Melbourne Longitudinal Study on Healthy Ageing (MELSHA) baseline cohort, offers a unique opportunity to examine prevalence and risk factors for self-reported medication side effects in an older cohort aged 65 years and over living independently in the community.

## Methods

### Study Design

The Melbourne Longitudinal Study on Healthy Ageing (MELSHA) is a prospective cohort study initiated in 1994. A cross sectional analysis was conducted on data collected at baseline in 1994. MELSHA was approved by the Human Ethics Committees of Latrobe University and Melbourne University.

### Study Population

The baseline MELSHA cohort, also known as the Health Status of Older People (HSOP) was comprised of a randomly selected community sample of 1000 English-speaking persons aged 65 years and over living independently in metropolitan Melbourne in 1994 [21,22]. A representative sample was randomly selected from 40 Melbourne postcodes using the electoral role (voting is compulsory for all Australian citizens between the ages of 18 and 70, and the names of all adults are maintained on the rolls, regardless of age) [22,23]. It is estimated that 95% of Australians over 80 years are on electoral role [23]. Those eligible comprised residents of private dwellings in metropolitan Melbourne. Potential participants were assessed by trained researchers by phone interview and face to face interview. Those excluded were those who lived in non-private accommodation such as nursing homes, hostels or hospitals (3.3%) or no longer living in metropolitan Melbourne (1.7%); those who could not be readily interviewed including those who did not speak basic English (11-12%), were cognitively impaired, were severely ill or had significant health problems (3-3.7%), were deceased (2.0%), or were deaf; and those with an incorrect date of birth (0.2%) [22,23]. Of the 1865 potentially eligible participants identified from the electoral role, 1422 were eligible [23]. Of those eligible 1000 participated, a response rate of approximately 70% [22,23]. Comparisons with the Census found that the sample was representative of older people in the Melbourne community with the exception of those too ill to be interviewed and non-English speaking people and that there was over-representation of home owners and those not relying on the pension as their main source of income [21,23].

### Data collection

Data were collected by questionnaire administered by face-face interview in participants' homes on a rolling basis from May to December 1994 [21-23]. Trained researchers interviewed participants using the HSOP questionnaire [21,22] and obtained self-reported answers to a wide range of demographic and social questions as well as details about active disease states, functional ability, and attitudes to health. There was verification of some self-reports by interviewer observation. A brief physical examination including height and weight and check of eye sight and hearing completed the interview [21,22]. Results presented in this paper are based only on data from the face to face interviews and survey. An extract of those questions used has been included as an Appendix.

### Risk factors: Exposures & Confounders

*Socio-demographic factors *included gender, age, marital status, education, and country of birth [21-23]. Age was collected as a continuous variable and then grouped as 65-74 and 75+ years. Country of birth was collected as categorical data including Australia, Great Britain or Ireland, Italy, Greece or Cyprus, Yugoslavia, Germany, Netherlands, New Zealand, Poland, South East Asia, Other European, and Other; and then classified as Australian and other. Education was measured as the highest attained education qualification and was collected as categorical data including Bachelor degree or higher, Trade or apprenticeship, Certificate or diploma and other; and then grouped as those with significant high school or further education and other. Marital status was collected as categorical variable including now married, living with a partner, widowed, divorced, separated, never been married and other; and then further classified as married or living with partner; or other (including widowed, divorced, separated, never been married).

*Health related variables *included, self-rated health a categorical variable scaled as excellent, very good, good, fair and poor; then collapsed as poor to fair, good and very good to excellent. Self reported use of hearing aids or glasses were measured as present or absent and were correlated with recorded interviewer observations about the use of glasses or contacts and use of hearing aid. Self reported medical conditions were measured by use of the Health Retirement Survey (HRS) format and a modified form of the Established Populations for Epidemiologic Studies of the Elderly (EPESE) and measured as present or absent [23]. The HRS asked if "the doctor has ever told you that you had [name of condition]" for blood pressure, heart attack or myocardial infarct, other heart problems including angina and heart failure, stroke, osteoporosis or brittle bones, and emotional, nervous or psychiatric problems. With the EPESE participants were shown a list of medical conditions and asked to indicate which conditions they had at the time including high cholesterol, low blood pressure, gout, diabetes or high blood sugar, osteoarthritis (arthritis unspecified), other types of arthritis ( including rheumatoid and other specified types), intestinal problems (such as ulcer or hernia), chronic bronchitis, emphysema, asthma, problems with feet and legs, cataracts, glaucoma, liver trouble or jaundice, kidney or bladder problems, anaemia, Parkinson's disease, migraine, eczema or dermatitis or psoriasis, skin cancer, other growths or cancer, varicose veins, thyroid disease, and any other condition [23]. There was no information collected from medical records or charts and no classification using the ICD system.

*Health service factors *included recency of visits to the General Practitioner (GP), pharmacist and checks of vision, hearing, and blood pressure classified as within a month, 1-5 months, 6-11months, 1-2 years, 3-4 years, 5 or more years, never. These were further grouped for visits to GP in the last month or more than a month ago; for visits and advice from the (chemist) pharmacy in the last 5 months or more than 5 months ago; checks of vision within the last 12 months, hearing within 5 years, and blood pressure within 1 month; and any hospital admissions in the last 12 months, recorded as Yes or No. Their doctor's gender and age was based on participant assessment and report of their doctor's age, and doctor's age was then grouped as younger or older than 50 years.

*Medication use *was based on self report of prescribed medications that had been taken or were supposed to have been taken in the 2 weeks preceding interview and recorded as Yes or No. There was no information available about the types of medications. The interviewer verified and checked medication containers and packaging that the participant had taken in the 2 week period prior to interview. Awareness of their usual doctor (General Practitioner (GP)) of their medications was based on participant assessment and report and was recorded as Yes or No. Use of strategies to assist in taking their medications was based on participant report and was recorded as Yes or No.

#### Outcomes

The outcome of interest was self-reported medication side effects ever up until the time of interview in 1994, and was recorded as Yes or No. There was no information collected about the severity or type of medication side effects.

#### Data Analysis

Data analysis was conducted on the whole cohort (n = 1000) to capture those who may have ceased taking medication due to side effects and on those currently taking at least one prescribed medication (n = 761). Preliminary analysis using chi square analyses tested direct relationships between independent variables (possible risk factors) and the dependent (outcome variable) of self-reported medication side effects. The association between existing risk factors including sociodemographics, health status and medical conditions, health service use and medication use and self-reported medication side effects was examined using logistic regression analysis. Univariate logistic regression was used to calculate crude odds ratios (OR) with 95% confidence intervals. Multivariate logistic regression analysis was used to calculate adjusted OR with 95% confidence intervals (95%CI). Factors achieving statistical significance at the bi-variate level (p < 0.20)in the univariate analysis, and/or documented in the literature or with plausible biological mechanisms were included in the multiple logistic regression model and included those risk factors described previously in the methods. Multi-collinearity was assessed by checking the partial correlation matrix of all factors introduced to the model. SPSS (*version 17*) was used for all statistical analysis.

## Results

### Sociodemographics

The baseline MELSHA cohort was predominantly aged 65 to 74 years, married, lacking in education and Australian born. There were similar numbers of males and females. See Table 1

**Table 1:** Socio-demographics, self reported health and medical conditions

		*n*	%	*N
*Sociodemographics *				
Age (years)	65 - 74	642	64	1000
	75+	358	36	
Gender	Male	467	47	1000
Country of Birth	Born in Australia	743	74	999
Marital Status	Married/living with partner	578	58	1000
Education	Left school less than 15 years or no qualification	591	59	997
*Health status &medicalconditions *Self-rated health				
	very good/excellent	484	49	988
	good	326	33	
	fair/poor	178	18	
Hypertension		429	43	996
Low Blood Pressure		37	4	995
High Cholesterol		124	12	995
Myocardial infarction		114	11	996
Other heart problem^a^		202	20	996
Stroke		58	6	996
Gout		67	7	996
Osteoporosis		68	7	996
Osteoarthritis		503	51	996
Other arthritis ^**b**^		61	6	996
Emotional/psychiatric problems		94	9	996
Thyroid disease		37	4	996
Diabetes		64	6	996
Intestinal problems		197	20	996
Liver problems		14	1	995
Kidney or bladder problems		117	12	996
Chronic bronchitis		62	6	996
Emphysema		44	4	996
Asthma		89	9	996
Cataracts		186	19	994
Glaucoma		53	5	994
Anaemia		36	4	995
Migraine		54	5	996
Parkinsons		7	1	996
Problems feet or legs		360	36	995
Varicose veins		210	21	996
Eczema, dermatitis, psoriasis		114	11	996
Skin cancer		112	11	995
Other growths or cancer		33	3	996

*N - total number of data for each variable

^a^Other heart problems including angina and heart failure

^b ^Other arthritis including rheumatoid and other specified types

### Health Status and Medical conditions

The baseline MELSHA cohort reported mostly good to excellent health. The most common self reported medical conditions included: osteoarthritis (51%), hypertension (43%), problems with feet or legs (36%), varicose veins (21%), intestinal problems (20%), other heart problems (20%), cataracts (19%), kidney or bladder problems (12%), high cholesterol (12%), skin problems (including eczema, dermatitis and psoriasis) (11%), skin cancer (11%) and emotional or psychiatric problems (9%). See Table 1. Furthermore, 15% of the cohort reported using a hearing aid and 96% reported wearing glasses.

### Health services

Most of the baseline MELSHA cohort reported they had a regular doctor (in Australia this is the role of the General Practitioner (GP)) (95%). Similar proportions reported seeing doctors older (49%) and younger (51%) than 50 years and most were male doctors (87.5%). Almost half the cohort (49%) reported that their blood pressure had been checked within 1 month, 42% reported that their vision had been checked within the last 12 months and 31% reported that their hearing had been checked within 5 years. Twenty two percent of the cohort reported that they had a hospital admission in the last 12 months. Most of the cohort reported that it had been less than a month since they had visited their doctor (60%), but more than 5 months since they had visited a pharmacist for advice (92%). Only 39% reported that their doctor (GP) was aware of all the medications that they were taking.

### Medication and Side effects

Of the baseline MELSHA cohort more than 76% reported currently taking at least one prescribed medications. Self-reported medication side effects were reported by approximately 6.7% (67/1000) of the entire baseline MELSHA cohort, and by 8.5% (65/761) of those on at least one medication.

#### Univariate analysis

Factors associated with an increased risk of self-reported medication side effects included: those whose doctors were aware of their medications; those with poor to fair self-reported health; and those with particular medical conditions such as osteoporosis, emotional or psychiatric problems, other arthritis including rheumatoid arthritis and other specified types, intestinal problems such as an ulcer or hiatus hernia, problems with feet and legs, liver problems, and skin problems (eczema or dermatitis or psoriasis). Mostly the risk of self-reported medication side effects was increased 2-4-fold, but for those with liver problems it was increased almost 6-fold. A similar pattern was seen for the baseline MELSHA cohort (entire population (n = 1000)) and those on at least one prescribed medication (n = 761). See Table 2

**Table 2:** Univariate Analysis Risk factors associated with self-reported medication side effects

	Entire population (*n *= 1000)			On medication (*n *= 761)		
		
	***OR***	95% *CI*	*p*	*OR*	95% *CI*	*p*
Recency doctor's visit^a^	0.49	0.28 - 0.86	0.013	0.77	0.43 - 1.36	0.365
Doctor aware medication^b^	3.11	2.44 - 3.96	< 0.0001	2.79	2.17 - 3.59	< 0.0001
Self reported health status ^c^	2.00	1.46 - 2.75	< 0.0001	1.65	1.19 - 2.27	0.002
Hypertension^d^	1.01	0.61 -1.67	0.971	0.63	0.37 - 1.05	0.075
Other heart problems^d ,^	1.89	1.10 - 3.24	0.021	1.48	0.85 - 2.55	0.163
Osteoporosis^d^	2.64	1.28 - 5.27	0.009	2.30	1.11 - 4.78	0.026
Emotional or psychiatric problems^d^	2.80	1.49 - 5.27	0.001	2.51	1.30 - 4.86	0.001
Other arthritis^d, f^	3.92	1.97 - 7.79	< 0.0001	3.02	1.47 - 6.18	0.003
Intestinal problem^d^	2.27	1.33 - 3.86	0.002	1.91	1.11 - 3.30	0.020
Problems feet or legs^d^	4.28	2.51 - 7.31	< 0.0001	3.64	2.12 - 6.26	< 0.0001
Liver problems^d^	5.83	1.78 - 19.11	0.004	6.45	1.84 - 22.63	0.004
Skin problems^d^	1.97	1.04 - 3.74	0.037	1.90	0.99 - 3.64	0.053

^a ^One month or more vs less than a month

^b ^Yes vs No; Doctor's awareness related to usual doctor (General Practitioner)

^c ^Very good to excellent vs poor to fair

^d ^Yes vs No

^e^Other heart problems including angina and heart failure

^f ^Other arthritis including rheumatoid and other specified types

#### Multivariate Analysis

The multivariate analysis predicted much of the risk of medication side effects with a pseudo R^2 ^of 0.442 (entire population) and 0.478 (on at least one prescribed medication). There were no strong correlations between the included risk factors as the largest partial correlations were 0.2-0.4.

Factors associated with an increased risk of medication side effects included: those with higher educational qualifications; attending younger doctors (< 50years); those who had last visited a pharmacist for advice more than 5 months ago; those who reported their doctor's awareness of their medications; those with particular medical conditions such as diabetes, problems with their feet or legs, skin problems (eczema, dermatitis or psoriasis) and cataracts. Mostly the risk was increased 2-4-fold. The greatest increased risk of 5-8-fold was for those who reported their doctor's awareness of their medications. The risk was decreased by approximately 75% for those with osteoarthritis and 65% for those with hypertension. A similar pattern was seen for the baseline MELSHA cohort (entire population (n = 536)) and those on at least one medication (n = 509). See Table 3

**Table 3:** Multivariate Analysis Risk factors associated with self-reported medication side effects*

	Entire population (*n *= 536)			On medication (*n *= 509)		
		
	*OR*	95% *CI*	*p*	*OR*	95% *CI*	*p*
Education^a^	2.66	1.07 - 6.65	0.036	3.43	1.26 - 9.30	0.016
Doctor's age^b^	2.38	1.01 - 5.56	0.048	2.70	1.08 - 7.14	0.035
Recency visit pharmacist for advice^c^	4.33	1.40 - 13.37	0.011	4.95	1.53 - 16.04	0.008
Doctor aware medication^d^	5.44	3.03 - 9.77	< 0.0001	7.83	3.83 - 16.01	< 0.0001
Osteoarthritis^d^	0.24	0.09 - 0.65	0.005	0.23	0.08 - 0.68	0.008
Hypertension^d^	0.41	0.16 - 1.04	0.059	0.34	0.12 - 0.93	0.035
Cataracts^d^	2.73	0.95 - 7.86	0.062	3.53	1.15 - 10.77	0.027
Diabetes^d^	3.01	0.82 - 11.06	0.096	4.00	0.98 - 16.82	0.053
Problems feet or legs^d^	3.87	1.57 - 9.53	0.003	3.97	1.51 - 10.46	0.005
Skin problems^d^	3.99	1.39 - 11.44	0.010	3.98	1.31 - 12.09	0.015

*Entire population pseudo R^2 = 0.442 (Nagelkerke); On medication, R^2 = 0.478 (Nagelkerke).

^a ^Completion high school or additional qualifications vs lower than high school education;

^b ^Aged younger than 50 years vs 50 years and older;

^c ^Five months or more vs less than five months;

^d ^Yes vs No

In the multivariate analysis, data was complete for 54% of the original cohort (n = 536) and 67% of those taking at least one medication (n = 509). Missing data mostly ranged from n = 1 to 7 and was greatest for participant reported doctor's age n = 249 and participant use of strategies for taking their medications n = 252. A comparison of each individual risk factors and outcome by missing status found that those with missing data tended to be in better health and were less likely to be on any medications. A sensitivity analysis in which the two risk factors with the greatest missing data participant reported doctor's age and participant use of strategies for taking their medications were excluded from the multivariate analysis for both the entire cohort (n = 915) and those currently taking medication (n = 710) gave a similar pattern of results with the loss of recency of visit to the pharmacist and addition of liver problems as seen in the univariate analysis.

## Discussion

The MELSHA study provided a unique opportunity to examine the prevalence and possible risk factors for self-reported medication side effects in a representative older cohort living independently in the community. The prevalence of self-reported medication side effects in our community based study was comparable with that seen in other studies in the primary care and community setting [2,3,24-28]. Our findings were consistent with prevalence estimates from the more recent BEACH reports (from General Practice based in a primary care community setting) 9.3% in 2007-2008 and 10.4% in 2003-2004 [27,28]. However, there was a marked disparity with prevalence estimates from hospital based studies through inpatients, outpatients and ambulatory or emergency departments of adults ranging in age from 18 to 75 years [8,16,29]. This disparity may reflect differences in the settings including selection, general health, use of medications and severity of medication side effects, i.e. those hospitalised or attending hospital are different to those living independently in the community attending General Practice. In addition, the disparity may also reflect differences in surveillance practices such as definitions and methods for detectionincluding measurement validity and sensitivity of exposures and outcomes.

The association with increased education of participants may reflect greater awareness and recognition of medication side effects through general knowledge, discussion with their doctor or pharmacist, and reading the product information. Recency of visiting the doctor within a month was protective and having last received advice from their pharmacist more than 5 months ago in contrast to less than 5 months ago was associated with an increased the risk of reporting medication side effects, lending support for the role of education in facilitating advice provided by doctors and pharmacists. In addition, it was suggestive that advice on medications may be forgotten over time by an older cohort, so the importance of regular monitoring and reinforcement by their doctors and pharmacists. The important educative role of doctors and pharmacists is further emphasised by another study that found starting a new medication, cessation of a medication or changes to prescribed and over the counter (OTC) medications were associated with an increased risk of medication side effects [14]. Therefore, doctors in prescribing, and pharmacists in dispensing, have an important role in detection and education.

The finding that an increased risk of medication side effects was associated with those with a younger treating doctor was more difficult to interpret as it was based on participants' assessment and report of their doctor's age and was associated with a high proportion of missing data. It may be that doctors who were assessed as younger tended to have less experience and make more therapeutic mistakes including dosing and interactions as has been suggested by another study [5]. Several studies have found that a significant proportion of medication side effects are preventable or ameliorable and include inadequate medical knowledge [13], flawed prescribing habits including the issuing of inappropriate scripts [13,30,31] and failure to monitor and review [13,30,31]. Alternately, younger doctors may be more likely to use newer medications for which side effects are not fully known or they may be more aware of medication side effects and/or better educate and raise their patient's awareness.

The association of medication side effects with their report of their doctor's awareness of their medications may reflect the use of prescription medication, recency of commencement of treatment, or use and monitoring of medications with low therapeutic/toxicity thresholds. Another possible explanation is that the prescribing doctor is aware, but other doctors involved in the patient's care are not, hence providing an opportunity for errors and interactions. Alternately, it may be that those who report their doctor's awareness of their medications are being monitored more frequently with greater opportunity to detect medication side effects.

The association of medication side effects with diabetes may reflect specific disease related issues around management including patient self management and collaboration of treating health professionals as well as therapeutic safety and/or greater opportunity for detection. In contrast for hypertension and osteoarthritis, there was a protective association with medication side effects. The difference in the findings for diabetes, hypertension and osteoarthritis, all chronic conditions likely to require regular follow up and review and hence offering the opportunity for detection, suggests that the opportunity for detection is an unlikely explanation. It may be that the difference in their findings reflects greater therapeutic safety of medications used in hypertension and osteoarthritis; more experience in their use by their usual doctor as management of hypertension and osteoarthritis is primarily managed by GPs in the primary care setting, whereas diabetes care may be more fragmented with management by specialists, GPs and other health professionals; or differences in patterns of associated co-morbidities and other factors not measured.

Self-reported poor to fair health was associated with an increased risk of medication side effects but it did not persist in the multivariate analysis when specific medical conditions were included. This association in the univariate analysis may have reflected significant medical conditions or alternately a focus on health including medication side effects. An increased risk of medication side effects was associated with particular medical conditions that may mimick medication side effects such as skin and liver problems; reflect changes to medication metabolism such as with liver problems especially in older patients; or use of medications with low therapeutic/toxicity thresholds [4,13,18]. The number with liver problems was too small to have sufficient power in the reported multivariate analysis. Cataracts were associated with an increased risk of medication side effects, possibly reflecting ability to read labels and instructions and comply. However, use of glasses or contacts was not associated with an increased risk, perhaps reflecting the use of a strategy to cope with restricted vision and hence ability to read labels. Other studies have found that medical conditions and patient related factors including impaired eye sight such as cataracts, hearing and cognition may impinge on compliance [13,32].

A lack of association with age and gender was consistent with the prevalence findings previously discussed; with a study that reported minimal changes in pharmaco-kinetics, pharmaco-dynamics and interactions with age [32]; and studies that have found that increasing age may be associated with increased numbers of medications [18,30,33,34] and multiple co-morbidities [17,18,33,34] which are independent risk factors. Those studies that have found an association between age and medication side effects [6,30,33,34], have been in young to middle aged patient groups [29] perhaps suggestive of an age threshold. As genetics may account for 20-95% variability in medication side effects [35], those who are long-lived may have favourable/protective genetics. Likewise, the lack of association with gender was consistent with those of another study in an older cohort with a mean age of 81.4 years in which no gender association was observed [34]. Again, those studies that have found an association with female gender [31,34,36] have been in young to middle aged patient cohorts [29].

There were several potential limitations of the study findings. The MELSHA data were collected in 1994 and may not reflect the current situation. However, the prevalence of self-reported medication side effects in the MELSHA cohort in the community in 1994 was/is similar to more recent and contemporary prevalence estimates of medication side effects in the BEACH reports. We have not focused on specific medications for which indications and use may have changed. Our analysis focused on independent stable risk factors including socio-demographics, health status and medical conditions, and health service factors. For example the measurement of age or classification of gender or marital status or medical conditions or definitions and scaling of self reported health status would be consistent now with 1994. The examined risk factors predicted a significant percentage (45%) of the risk of self-reported medication side effects.

The reliance on recall and self report may have potentially introduced systematic error or bias affecting the internal validity of the study findings as well as random error affecting the reliability and precision of the study findings. The internal validity of the study may have been affected by information bias due to the reliance on recall and self report. Such a possibility can not be definitively excluded. However, previous studies have found good levels of agreement between recall and self report of medication usage with pharmaceutical claims data as well as home visit verification [37,38]. It is possible that the prevalence of medication side effects may have been underestimated as only more recent or severe or persistent medication side effects may be recalled. However, our findings with respect to the prevalence of medication side effects appeared to be consistent with those from other studies including the more contemporary studies in the ongoing BEACH report as discussed previously [27,28]. It is also possible that those with medication side effects may have reflected more on possible associated risk factors. However, socio-demographic factors would not be affected and those of medical conditions and medications were verified independently by the interviewer. The survey was administered by a trained interviewer and so there was the opportunity for clarification and probing. In addition, the interviewer provided a standard written list of medical conditions and checked medications, providing some additional verification to self-report alone with respect to medical conditions and medications. Therefore, although recall bias is a possibility, it would seem an unlikely explanation for our findings. In addition, the effect of potential bias from potential confounders has been adjusted for in the multivariate analysis. However, it is not possible to adjust for unknown confounders or confounders on which data were not collected.

It has been suggested that self report of medication side effects may be less accurate and less reliable than chart review that is possible in clinical settings [32,34]. A lack of reliability in the use of self-report, may result in lack of precision and loss of power to detect an effect/association. However, a study of older Australians' medication use that examined self report by phone compared with a home visit and inventory found self report was accurate (high agreement as measured by kappa) for all prescribed medication categories [37].

Other potential issues were missing data and the small numbers with medication side effects. Missing data may have reduced the power to detect an association or introduced potential bias. However, sensitivity analyses around missing data found a similar pattern of results. The small numbers with medication side effects may have meant that the study had insufficient power to examine risk factors of smaller magnitude of effect or rarer/less common risk factors, but we have only commented on those associations detected by this study. The small numbers also meant that some categories had to be collapsed which may have further affected the findings, although this was limited by an apriori approach based on similarity and logic.

Overall, given the random sampling and selection of the MELSHA cohort, the findings are likely to be generalisable to older people living independently in the community. It is important to reflect on strategies to address some of the potential risk factors identified. From a review and framework proposed by the National Prescribing Service (NPS) [39] in conjunction with our findings, key approaches would include: targeting specific diseases identified by our study; addressing the awareness and knowledge of health professionals in particular doctors and pharmacists as well as patients; and communication between health professionals and between health professionals and patients [39].

In targeting specific diseases diabetes would be a useful starting point, and other diseases that may be considered include osteoporosis, heart problems such as arrhythmias and cardiac failure, emotional or psychiatric conditions and other arthritis. In addition to existing strategies for improved diabetes management, it may be that the management of hypertension and osteoarthritis may provide insights into disease focused management strategies to minimize medication side effects. As well, the need for additional care in using medications in those with liver problems needs emphasising.

Greater awareness and knowledge of health professionals including doctors and pharmacists as well as patients is also important. Further education during medical training and scaffolding in the workplace may support doctors in developing safe rational prescribing practices that minimize medication side effects. Specific education strategies for health professionals in particular doctors and pharmacists may include integration into undergraduate curriculae as well as post graduate/professional ongoing continuing medical education programs; supportive systems such as provision of information through professional colleges, support software, and emphasis on continuity of care over fragmentation; and quality control through participation in audits. In particular areas needing highlighting include the need for increased awareness and follow up and review in relation to the timing of medication side effects such as when introducing new medications and in particular for newly licensed medications as well as over the counter medications; similarly with the cessation and changing of medications; increased awareness of the range of presentations of medication side effects and the need to consider as a differential diagnosis; importance of participation in national surveillance and reporting systems for adverse drug reactions to increase the shared information available and the ability to detect a signal earlier. Improved health education of patients may enable early recognition and prevention of potential medication side effects.

Communication between health professionals including between GPs and specialist as well as pharmacists and other health professionals and with patients may facilitate management as well as surveillance and detection. Given the pivotal role of treating doctors and pharmacists in providing patient education and monitoring/surveillance of medication side effects, factors such as continuity or systems for information sharing may facilitate this role for doctors and pharmacists as well as other health professionals. As suggested by the NPS e-health may be one way in which such communication and information sharing may occur.

Finally in recognising and better managing medication side effects, it is important that medication side effects are not simply attributed to increased age. This requires increased awareness and consideration of other potential risk factors in older people, to better prevent and manage medication side effects. Prevention may require greater use of strategies by patients and health professionals to address potential sensory, physical and cognitive deficits with aging that may be associated with compliance problems: ongoing supervision and use of dosette boxes; regular medication reviews; provision of readily understood written instructions on medications including how to take and potential side effects in large readily read print; establishment of hotlines for patients to obtain additional information this would offer the potential to provide for those with hearing or language difficulties.

## Conclusion

The findings from the MELSHA cohort are generalisable to older people living independently in the community. Identified risk factors provide insights and directions for strategies to address the important issue of reducing medication side effects in older people. In the future, it will be important to develop education programs and policy that improve the level of awareness of medication side effects and provide greater opportunity for prevention and early management.

## Competing interests

All authors declare that there are no competing interests and therefore have nothing to declare.

The Corresponding Author has the right to grant on behalf of all authors and does grant on behalf of all authors, an exclusive licence.

## Authors' contributions

JAT contributed to the conceptualization and design of this specific sub-study, data extraction and analysis and interpretation and drafting the manuscript. WCW contributed to data extraction and analysis and commented on the manuscript. CB was and is the joint leader of the MELSHA project and participated in its design and co-ordination and funding acquisition, provided data and commented on the manuscript. HLK was and is the joint leader of the MELSHA project and participated in its design and co-ordination and funding acquisition, provided data and commented on the manuscript. All authors have read and approved the final manuscript.

## Appendix Summarised Extract of Questions from Survey Instrument

### 1. Interviewer administered survey

#### Background Socio-demographic information

"Record gender (do not ask): Male, Female"

"How old are you (age in years"

"In what country were you born: Australia, Britain or Ireland, Italy, Greece or Cyprus, Yugoslavia, Germany, Netherlands, New Zealand, Poland, South East Asia, Other European, Other(specify), Don't know/refused)"

"Which of these best describes your highest qualification: Bachelor degree or higher, Trade/apprenticeship, certificate/diploma, Other (specify), Don't know/refused."

"Ask only if necessary: Are you now married (Probe as needed to code further): Now married, living with partner, widowed, divorced, separated, never been married, other (specify), don't know/refused."

#### Health conditions: Health status & Self-reported medical conditions

##### Health status

"Now I have some questions about your health. Would you say that for someone of your age your own health, in general, is (*Read Out*): Excellent, Very Good, Good, Fair, Poor, Don't know/refused."

##### Medical conditions

"Has a doctor **ever **told you that you have (*Read out each condition ...........code as Yes or No*): blood pressure, heart attack or myocardial infarct, other heart problems, stroke, osteoporosis or brittle bones, emotional, nervous or psychiatric problems."

"Now I want to show you a list of other medical conditions. Please tell me which of these medical conditions you have now? ( Show card with list of medical conditions ..........code as Yes or No): high cholesterol, low blood pressure, gout, diabetes or high blood sugar, osteoarthritis (arthritis unspecified), other types of arthritis ( including rheumatoid and other specified types), intestinal problems (such as ulcer or hernia), chronic bronchitis, emphysema, asthma, problems with feet and legs, cataracts, glaucoma, liver trouble or jaundice, kidney or bladder problems, prostate trouble (men only), anaemia, Parkinson's disease, migraine, eczema or dermatitis or psoriasis, skin cancer, other growths or cancer, shingles, varicose veins, and thyroid disease".

Note in the analysis prostate problems was omitted as gender specific and shingles was omitted as short term episodic.

##### Use of aids

"Next I want to ask you about aids or equipment you may use. Do you currently use any aids or equipment on this card? (Read out each item if necessary..............code 1 for used and 0 if not used):....hearing aid, ...., glasses or contact lenses?"

#### Health services

"Have you been admitted to Hospital in the last 12 months: Yes, No, Don't know/refused."

"Using this card ( show card.....). Please tell me about how long is it since you've: (Read out below: Codes-within a month, 1-5 months, 6-11 months, 1-2 years, 3-4 years, 5 or more years, never): seen a doctor, ....................been to a chemist for advice?"

"About how long is it since you've (Read out) use codes from previous card: Codes-within a month, 1-5 months, 6-11 months, 1-2 years, 3-4 years, 5 or more years, never): ......................., Had your blood pressure checked?,................."

#### Medications

"The next few questions are about medicines. Could you please show me any medications prescribed by a doctor that you have taken or were supposed to take in the last 2 weeks.................... Check the prescribed medicines against the medications list............., recorded as No or Yes".

"Does the respondent have any medicines ................prescribed by a doctor that he/she has taken or was supposed to take in the last two weeks? Yes, No, Don't know/refused"

"Was this prescribed by your doctor? Circle "1" if answer is yes."

"Does your doctor know you use this medication? Circle "1" if answer is yes."

"Do you have any side effects from this medication? Circle "1" if answer is yes".

"Do you do anything to make sure you take the right amount of medicine at the right time? Code Yes, No, Don't know/refused."

### 2. Interviewer Observations

Observations on gender, ability to speak English, physical difficulties including hearing impairment, crippled hands or legs, understanding of questions, difficulty remembering and accuracy of responses

### 3. Self completion survey

Do you have a regular doctor?

About how old is your doctor (age in years)?

Is your doctor a man or woman?

## Pre-publication history

The pre-publication history for this paper can be accessed here:

http://www.biomedcentral.com/1471-2318/10/37/prepub
